# The Vaccination Act

**Published:** 1903-03-14

**Authors:** 


### The Hospital.

A Journal of

The Medical Sciences and Hospital Administration.

Vol. XXXIII.—No. 859. Edited by Sir Henry Burdett, K.C.B., and Solomon C. Smith, M.D., M.R.C.P. [March 14, 1903.

### The Vaccination Act.

In reply to a question by Mr. Coghill in the House of Commons, the Prime Minister, on the 4th instant, declared it to be the intention of the President of the Local Government Board to renew the existing Vaccination Act for this year, and to defer any further legislation on the subject to a future session of Parliament. He declined to pledge himself as to whether the Act would be renewed for one year only, or for a longer period, but expressed an opinion that the former would be the more convenient course; and, in reply to a further question, said that he was "not aware" of there being serious outbreaks of small-pox in several large towns like Manchester and Liverpool. It of course might easily happen, if the facts were as implied in the question, that the Prime Minister would not "be aware" of a circumstance lying outside the immediate cognisance of his department, and that the answer was not intended as an explicit negation, but only as a frank admission of want of knowledge on the matter referred to. Still, the question itself would almost justify the inference that the honourable member putting it was himself in possession of definite information which led him to expect a reply in the affirmative, more especially when it is considered that he represents "a large town" to which his inquiry might be supposed to apply. It is therefore consolatory to find that the weekly report of the Registrar-General, on the births and deaths in London and 75 other great towns, which had been made public on the very day of Mr. Coghill's inquiry, does not appear to furnish any occasion for alarm. Seven deaths from small-pox had been registered during the week in Liverpool, four in Manchester, and one each in Aston Manor, Birkenhead, Bootle, Oldham, Burnley, and Preston; but not one among Mr. Coghill's constituents at Stoke-upon-Trent, nor in any other of the 76 towns. Seven deaths in Liverpool do indeed furnish a sufficient reason for increased vigilance and activity on the part of the local health authorities, and for strenuous endeavours to obtain the vaccination of the unprotected members of the juvenile population; but in a city containing something like three-quarters of a million of people they hardly represent anything which deserves to be called a "serious outbreak."

Mr. Long's decision to renew the Vaccination Act as it stands for at least another year, and thus to defer legislation until time has been afforded for careful consideration of the amendments of the law which are required in the interests of the public safety, and which will be the least likely to afford ground for factious or foolish opposition, is one which will generally commend itself to the judicious. We fear it must be admitted that there are vaccination as well as anti-vaccination fanatics, and that the former, in the intensity of their convictions as to what is right, are apt to lose sight of the equally important question of what is possible or expedient. It is a matter for public congratulation that the question is in the hands of Mr. Long, whose entire belief in the efficacy of vaccination and re-vaccination, and in the desirableness of rendering them universal, has been declared in no uncertain language, and whose disregard of mere ignorant clamour has been shown, in a very striking manner, by his firmness in respect to rabies; but who is at the same time a statesman, conversant with the strength of the forces against which he has to contend, and trained in the practice of cutting away the ground from under the feet of his opponents. We know that, in his own incisive words, he proposes to commit the gradual elimination of the "conscientious objector" to the Board of Education; and that, pending the completion of the process, he is likely to seek, by improved methods of administration, the removal of all impediments to vaccination which are essentially of an administrative character. It is practically certain that any future Bill on the subject will aim at removing the control of the process from Boards of Guardians and their officers, in order to commit it to those sanitary authorities who are charged with the general duty of safeguarding the public health. Nothing can be more certain than that the influence of the anti-vaccinators, like that of their congeners the anti-vivisectionists, has been chiefly due to the density of the popular ignorance to which they have been able to appeal, and to the absence of any systematic endeavour to oppose falsehood by truth. The single fact to which we briefly called attention last week, that while Purfleet had 53 cases of small-pox among a population of 600, manifestly as a result of the neighbourhood of the hospital ships moored in Long Reach, not a single case occurred among the united populations of the Purfleet garrison and of the *Cornwall* training ship, amounting to 467 persons, all of whom were equally exposed to the infection, but who had all been effectively vaccinated, is alone sufficient, if it were pressed home to the public mind as it might be, and ought to be, to dispose of nearly all the falsehoods by which poor and simple folk have been beguiled.

